# Efficacy and safety of combination immunotherapy and targeted therapy as second-line and subsequent treatments for advanced biliary tract cancer: A systematic review and meta-analysis

**DOI:** 10.1097/MD.0000000000049290

**Published:** 2026-06-19

**Authors:** Yiran Liao, Zhongli Liao, Jiong Wang

**Affiliations:** a Phase I Clinical Research Center, Chongqing University Cancer Hospital, Chongqing China; b Department of Gastroenterology, Chongqing University Cancer Hospital, Chongqing, China.

**Keywords:** biliary tract cancer, efficacy, immunotherapy, meta-analysis, targeted therapy

## Abstract

**Background:**

Biliary tract cancer is a highly dangerous cancer that often results in a poor prognosis for patients, even with significant advancements in treatment options. Recent advancements in targeted therapies and immunotherapy have shown great potential in treating various cancers. This underscores the importance of conducting a detailed review and meta-analysis of these approaches specifically for biliary tract cancer.

**Methods:**

This meta-analysis included data from randomized clinical trials and cohort studies examining the use of immunotherapy and targeted therapies specifically as second-line treatments for patients diagnosed with biliary tract cancer. A comprehensive andsystematic literature search was conducted across prominent databases, including PubMed, Embase, Cochrane Library, and Web of Science, encompassing publications from the inception of these databases until August 31, 2024. Key outcome measures, such as objective response rate (ORR), DCR, progression-free survival, overall survival, and AEs, were meticulously extracted for analysis.

**Results:**

The findings of this meta-analysis revealed that the ORR across the included studies varied from 4% to 33%, with a combined ORR of 14% (95% confidence interval [CI]: 9%–19%). The pooled DCR was calculated at 68% (95% CI: 53%-77%). Median overall survival was reported as 10.75 months (95% CI: 8.23–13.27 months), while median progression-free survival was 4.39 months (95% CI: 2.73–6.04 months).

In terms of AEs, most patients experienced only grade 1 to 2 events, indicating a generally favorable tolerance to treatment. The most commonly reported AEs included fatigue (44%, 95% CI: 31%–58%), hypertension (41%, 95% CI: 28%–55%), and nausea (23%, 95% CI: 18%–29%). Additionally, thrombocytopenia emerged as the most prevalent hematological toxicity, occurring in 31% of patients (95% CI: 23%–39%). Notably, the incidence of ≥grade III AEs was very low, remaining below 14%. Among the most frequent severe AEs were hypertension and elevated aminotransferases, with incidences of 14% and 8%, respectively.

**Conclusion:**

This comprehensive meta-analysis highlights the potential role of targeted therapies and immunotherapy in the management of biliary tract cancer, demonstrating promising albeit moderate efficacy. These findings underscore the necessity for further research and clinical trials aimed at optimizing treatment protocols to enhance outcomes for patients afflicted with this challenging malignancy.

## 1. Introduction

Biliary tract cancer (BTC) encompasses a heterogeneous group of malignant tumors, including gallbladder cancer (GBC), intrahepatic cholangiocarcinoma (iCCA), and extrahepatic cholangiocarcinoma (eCCA), further categorized into hilar and distal cholangiocarcinoma. BTC accounts for approximately 3% of all gastrointestinal cancers and 10%–15% of primary liver malignancies, making it the second most common liver cancer after hepatocellular carcinoma.^[1–3]^ Recent epidemiological studies reveal a concerning rise in BTC incidence, particularly in endemic regions such as China, where rates are approximately 40 times higher than those in high-income countries.^[1–3]^

The prognosis for BTC patients is dismal, with nearly 50% presenting with advanced-stage disease, leading to a median survival of only 3 to 6 months.^[1,3,4]^ The 5-year survival rates for stages III and IV BTC are alarmingly low, at 10% and 0%, respectively. Surgical interventions are viable for only about 10% of patients, with postoperative recurrence rates exceeding 67% within the first year.^[3,4]^ In 2010, the ABC-02 trial^[5]^ established the combination of gemcitabine and cisplatin as a significant improvement over single-agent gemcitabine, extending the median overall survival (OS) of patients with advanced BTC to 11.7 months, thereby solidifying GC as the standard first-line treatment for this population. Following the ABC-02 trial findings, 15% to 25% of patients continue to receive subsequent therapy after failing the CisGem regimen, with this percentage likely higher in real-world clinical practice for patients without obstructive jaundice. However, the past decade has seen relatively limited progress in the treatment of advanced BTC, with no high-level evidence supporting the clinical benefits of second-line chemotherapy, nor any standardized chemotherapy regimens recommended. In 2019, various guidelines, based on the results of the open-label, multicenter, phase II randomized controlled trial ABC-06 trial,^[6]^ recommended “active symptom control (ASC) + mFOLFOX (oxaliplatin + 5-FU)” as a second-line treatment option for patients with advanced BTC who have failed the CisGem regimen. Despite these advancements, the efficacy of treatments for advanced refractory BTC remains inadequate, underscoring the urgent need for innovative therapeutic strategies to improve patient outcomes.

BTC is characterized by a complex genetic landscape, with actionable mutations identified through next-generation sequencing, including fibroblast growth factor receptor (FGFR) fusions and isocitrate dehydrogenase (IDH) mutations.^[7]^ The emergence of immunotherapy, particularly immune checkpoint inhibitors (ICI), has shown promise across various malignancies.^[8–10]^ However, studies in BTC reveal limited efficacy, with response rates ranging from 3% to 11% and median progression-free survival (PFS) of 1.4 to 3.7 months and median OS of 5.2 to 14.2 months.^[11]^ Notably, the durability of responses, often extending beyond 6 months, is of particular interest.

The combination of immunotherapy and targeted agents represents a promising new frontier in the treatment of advanced BTC. Evidence suggests that anti-angiogenic drugs can reduce immunosuppression, while immunotherapies can have anti-vascular effects, creating a potential virtuous cycle of immune stimulation and vascular remodeling within tumors.^[12]^ Several recent studies investigating the combinatorial use of angiogenesis inhibitors with ICIs have yielded promising results. For instance, the combination of lenvatinib and pembrolizumab demonstrated a response rate of 10%, with a median PFS of 6.1 months and a median OS of 8.6 months. Moreover, emerging data from early-phase trials indicate that combinations such as toripalimab plus lenvatinib and camrelizumab plus apatinib may provide enhanced clinical benefits compared to monotherapy, with reported median OS outcomes ranging from 8.6 to 13.1 months.^[13]^

Despite these encouraging findings, the majority of existing trials are non-randomized and characterized by small sample sizes, making the generalizability of their outcomes limited.^[3,14–20]^ As a result, rigorous evaluations of the safety and efficacy of these combinatory approaches are essential to substantiate their clinical utility. Consequently, the objective of this meta-analysis is to systematically evaluate the efficacy and safety of immunotherapy combined with targeted therapies as second-line and subsequent treatment options for advanced biliary tract malignancies. We anticipate that the findings from this analysis will provide critical insights that can inform the development of more effective clinical strategies for managing these challenging conditions, ultimately improving patient outcomes in this high-need area of oncology.

## 2. Materials and methods

### 2.1. Search strategy

This systematic review and meta-analysis were conducted and reported in accordance with the Preferred Reporting Items for Systematic Reviews and Meta-Analyses guidelines. It has been registered with PROSPERO under registration number CRD42024570038.

We conducted a thorough search for pertinent studies across 4 key databases: PubMed, Embase, Cochrane Library, and Web of Science. The most recent search was performed on August 31, 2024, and was limited to English-language publications. Additionally, we explored the Chinese databases CNKI and SINOMED but did not find any further relevant studies in Chinese. To enhance the comprehensiveness of our search, we also reviewed resources from the European Society of Medical Oncology, the American Society of Clinical Oncology, and the Chinese Society of Clinical Oncology. Furthermore, we meticulously examined the reference lists of the included articles to identify any additional relevant studies.

The following MeSH terms and free-text keywords were used in the searches: (“Biliary Tract* Neoplasm” OR “Biliary Tract Cancer*” OR “Cholangiocarcinoma” OR “eCCA” OR “GBC”) AND (“Immunotherapy” OR “Immunotherapies” OR “Immune checkpoint inhibitor*” OR “Immune Checkpoint Blockers” OR “Durvalumab” OR “Pembrolizumab” OR “Nivolumab”) AND (“Targeted therapy” OR “Molecular Targeted Therapies” OR “VEGFR” OR “EGFR” OR “NTRK” OR “IDH” OR “PARP” OR “BRAF” OR “HER2”).

### 2.2. Inclusion and exclusion criteria

The inclusion and exclusion criteria for this study were carefully developed based on the PICOS framework. We concentrated on randomized controlled trials and cohort studies. To be eligible for inclusion in this meta-analysis, studies needed to meet the following criteria:

Population: The specific population considered for analysis included patients with pathologically confirmed advanced or recurrent cholangiocarcinoma, encompassing both intrahepatic and extrahepatic types, who exhibited resistance to systemic first-line therapy.

Intervention: In terms of interventions, only studies involving patients receiving ICI and targeted therapies concurrently were included. The specified therapies comprised programmed cell death protein 1 (PD-1) inhibitors, PD-L1 inhibitors, VEGFR-2 antagonists, MEK inhibitors, and multi-target therapies (such as those targeting VEGFR and FGFR).

Outcomes: Furthermore, eligible studies were required to report on relevant clinical tumor outcomes, including the objective response rate (ORR), disease control rate (DCR), PFS, OS, and AEs. Tumor responses were assessed according to the Response Evaluation Criteria in Solid Tumors version 1.1. Adverse effects were evaluated based on their incidence and severity using the Common Terminology Criteria for Adverse Events.

Exclusion Criteria: The exclusion criteria were comprehensively defined to retain only studies that provided the most complete and current data, particularly in cases where data originated from different phases of the same experimental study.

### 2.3. Data extraction and quality assessment

In this study, data were independently extracted by 2 investigators to ensure both accuracy and reliability. The extracted variables were systematically summarized and included the following: author, year of publication, country of origin, sample size, number of prior treatment lines, median age of participants, Eastern Cooperative Oncology Group performance status score, and median duration of follow-up.

The clinical and safety outcome measures evaluated encompassed the ORR, DCR, OS, PFS, as well as the incidence of any AEs and those classified as grade ≥3 AEs.

To assess the methodological quality of the noncontrolled trials included in the analysis, we employed the Newcastle-Ottawa Scale (NOS) as a standardized evaluation tool.^[21]^

### 2.4. Statistical analysis

We utilized STATA for the meta-analysis. Heterogeneity was assessed using the *I*^2^ statistic, where a *P*-value of <.1 was considered indicative of a statistically significant difference. In cases where significant heterogeneity (*P*-value <.1 and *I*^2^ > 50%) was observed, we applied a random-effects model; otherwise, a fixed-effects model was implemented.^[22,23]^

### 2.5. Ethical considerations

This study is a systematic review and meta-analysis based exclusively on previously published data. No individual patient data were collected, and no human participants were directly involved. Therefore, approval from an institutional review board or ethics committee was not required, in accordance with international guidelines for secondary research.

## 3. Result

### 3.1. Study selection

**Figure 1.**
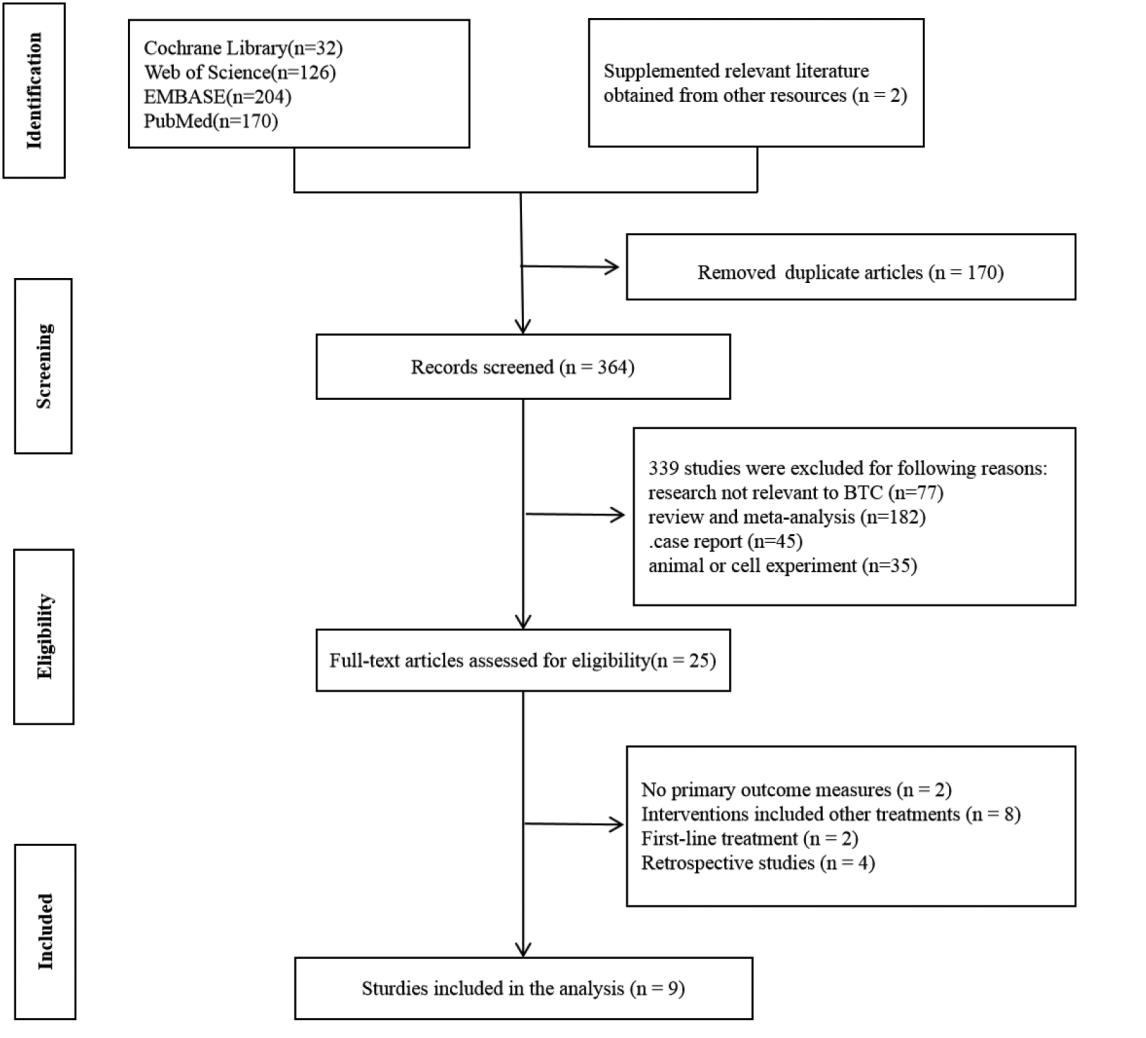
Flowchart of study selection.

A total of 534 published studies were obtained from 4 databases (Cochrane Library (n = 32), Web of Science(n = 126), EMBASE(n = 204), PubMed(n = 170)). After removing duplicates and sifting through titles and abstracts, 25 studies were retained. The remaining full-text articles were then evaluated one by one, with 2 studies having no outcome measures, 8 studies involving other treatments, 2 studies being first-line treatment, and 4 retrospective studies. Ultimately, 9 studies with a total of 371 patients met the inclusion criteria and were included in this meta-analysis. The flow chart of the selection process is shown in Figure 1.

We included 9 studies in this analysis, all focusing on targeted therapies combined with PD-1/PD-L1 monoclonal antibodies. The studies consist of 5 phase II trials, 2 phase I/Ib trials, and 2 prospective cohort studies, all registered clinical trials published between 2018 and 2023, with the earliest initiated in 2017 (Table 1).

**Table 1:** Characteristics of the studies included in the meta-analysis.

Study	Registration number	Phase	Sample size	Tumour location	ECOG	Prior regimens	Median age, yr	Median follow-up, mo	Intervention	NOS
Luis(2021)^[20]^	NCT03797326	II	31	NR	0:15 1:16	1	NR	NR	Pembrolizumab 200 mg d1 IV Q3W/ Lenvatinib 20 mg d1–21 PO Q3W	6
Jin(2023)^[15]^	ChiCTR1900022003	II	20	ICC11/ECC3/GBC6	0:14 1:6	1	58 (43–69)	12.5 (7.1–21.8)	Sintilimab 200 mg d1 IV Q3W/ Anlotinib 12 mg d1–14 PO Q3W	7
Ueno(2024)^[14]^	jRCT2091220436	II	32	ICC15/ECC5/GBC7/ Ampulla of Vater5	0:23 1:9	1	63 (44–78)	6.4	Nivolumab 240 mg d1 IV Q2W/ Lenvatinib 14–20 mg d1–14 PO Q2W	6
Zhou(2023)^[11]^	NCT03825705/ NCT03996408	Ib	66	ICC31/ECC15/GBC20	0:43 1:23	1	58 (35–75)	19.68 (16.33–20.44)	TQB2450 1200 mg d1 IV Q3W/ Anlotinib 10–12 mg d1–14 PO Q3W	7
Arkenau(2018)^[3]^	NCT02443324	I	26	GBC/CCA	0:12 1:14	1–2	63 (36–78)	15.7 (10.3–17.0)	Pembrolizumab 200 mg d1 IV Q3W/ Ramucirumab 8 mg/kg d1 d8 IV Q3W	7
Cousin(2022)^[17]^	NCT03475953	II	34	ICC26/ECC7/GBC1	0:16 1:17 NR:1	≥1	63.1 (36–80)	9.8 (6.6–12.4)	Avelumab 10 mg/kg d1 d15 IV Q4W/ Regorafenib 160 mg d1–21 PO Q4W	7
Yarchoan(2021)^[19]^	NCT03201458	II	38	ICC8/ECC22/GBC8	0:8 1:30	1–2	<50 (4) 50–70 (24) >70 (10)	NR	Atezolizumab 840 mg d1 d15 IV Q4W/ Cobimetinib 60 mg d1–21 PO Q4W	8
Chao(2023)^[16]^	NCT03892577	Cohort	103	NR	0:NR 1:NR 2:NR	≥1	57 (30–82)	17.3 (2.3–37.3)	Pembrolizumab/Tislelizumab/Sintilimab/Camrelizumab 200 mg or Toripalimab 240 mg d1 IV Q3W/ Lenvatinib 8–12 mg d1–21 PO Q3W	7
Wang(2021)^[18]^	NCT04642664	Prospective	21	ICC15/ECC4/GBC2	0:2 1:16 2:3	≥1	60 (39–72)	13.4 (11.9–14.8)	Camrelizumab 200 mg d1 IV Q3W/ Apatinib 250 mg d1–21 PO Q3W	7

NR = not reported.

Notably, 2 of these studies (Luis(2021),^[20]^ Chao(2023)^[16]^) did not specify the locations of the tumors studied. In contrast, 5 studies (Zhou(2023),^[11]^ Wang(2021),^[18]^ Yarchoan(2021),^[19]^ Cousin(2022),^[17]^ Jin(2023)^[15]^) identified these tumors as ICC, ECC, and GBC. Additionally, Arkenau(2018)^[3]^ reported tumors classified as GBC and CCA, while Ueno(2024)^[14]^ specifically detailed occurrences of ICC, ECC, GBC, and tumors located at the ampulla of Vater. (Table 1)

The patient cohort across the 9 studies included 106 cases of ICC, 56 cases of ECC, 44 cases of GBC, and 5 cases of ampullary tumors. Notably, 3 studies involving 160 patients (Arkenau(2018),^[3]^ Luis(2021),^[20]^ Chao(2023)^[16]^) did not explicitly classify the types of biliary tract cancers among the participants. Sample sizes varied from 20 (Jin(2023)^[15]^) to 103 participants (Chao(2023)^[16]^). Two studies (Luis(2021),^[20]^ Yarchoan(2021)^[19]^) did not report follow-up data, while the remaining 7 studies indicated a median follow-up duration ranging from 6.4 months (Ueno(2024)^[14]^) to 19.68 months (Zhou(2023)^[11]^). Importantly, baseline characteristics revealed a significant prevalence of lower Eastern Cooperative Oncology Group performance status scores, with the majority of participants scoring between 0 and 1, indicating that they generally maintained good functional status. The median age of participants ranged from 35 to 82 years. (Table 1) Treatment regimens primarily consisted of ICIs, including pembrolizumab, nivolumab, and sintilimab, often administered in conjunction with targeted therapies such as lenvatinib and regorafenib. (Table 2)

**Table 2:** Primary target sites in targeted and immunotherapy studies.

Primary target sites	Specific drug
PD-1	Pembrolizumab, Toripalimab, Tislelizumab, Sintilimab, Camrelizumab, Nivolumab
PD-L1	Atezolizumab, Avelumab, TQB2450
VEGFR-2	Ramucirumab, Apatinib
MEK	Cobimetinib
Multi targets	Lenvatinib, Regorafenib, Anlotinib

### 3.2. Quality assessment

Studies were assessed according to the NOS, which evaluates 3 critical dimensions: selection of participants, comparability, and outcome or exposure assessment. The NOS employs a semiquantitative star rating system, with a maximum possible score of 9 stars; higher scores reflect superior study quality. Among the 9 studies reviewed, 2 achieved a score of 6, one received a score of 8, and the remaining 6 studies were rated 7 (Table 1). Consequently, the overall quality of the included studies was classified as high.

### 3.3. Tumor response

**Figure 2.**
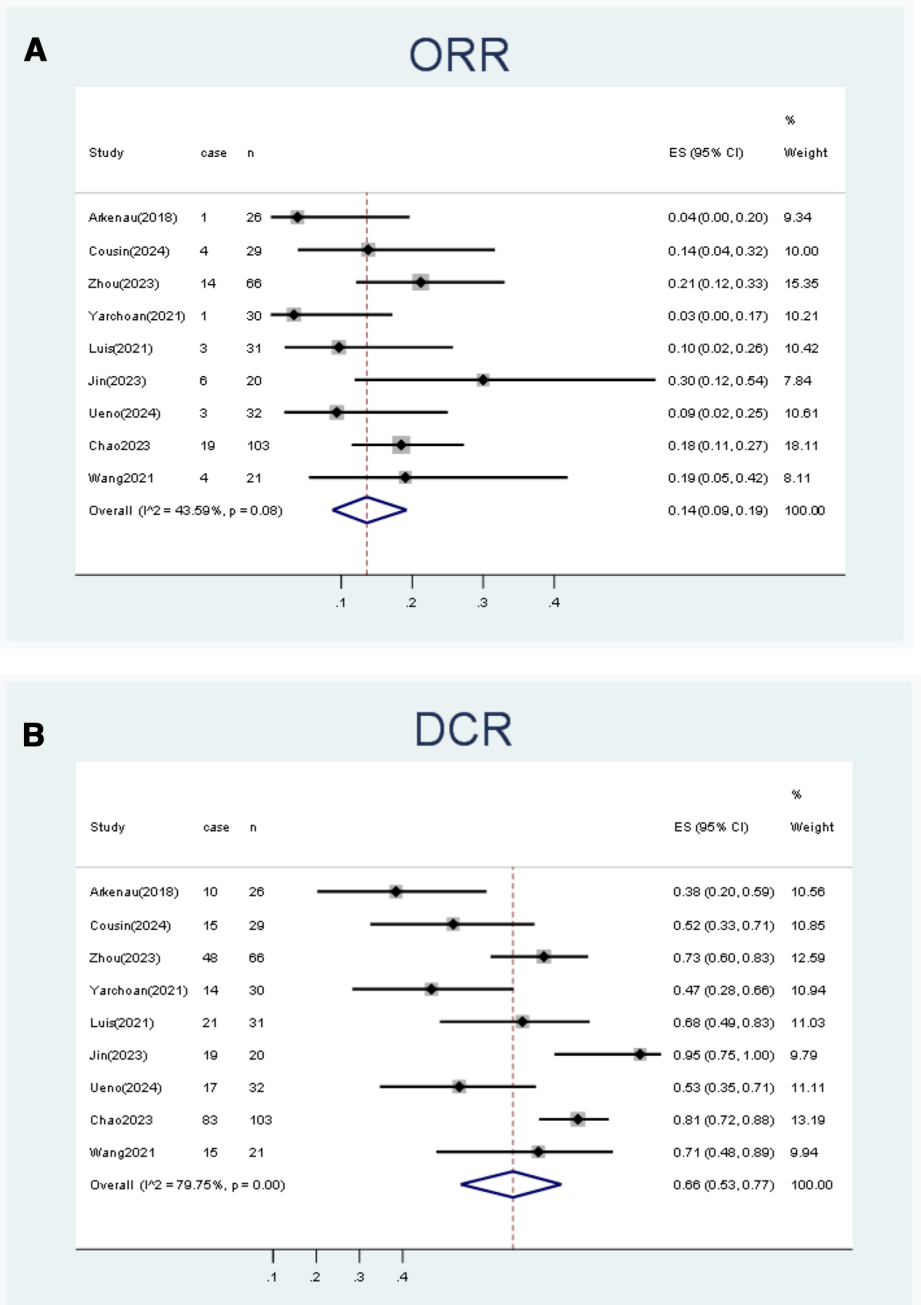
Forest plot about the pooled results of ORR (A) and DCR (B) in total by the treatment regimen subgroup. DCR = disease control rate, ORR = objective response rate.

All studies included in this review demonstrated the efficacy of targeted combination immunotherapy in biliary malignancies that had been previously treated with at least one systemic first-line therapy. The ORR across these studies ranged from 3% (Yarchoan(2021)^[19]^) to 30% (Jin(2023)^[15]^). Due to significant heterogeneity, a random effects model was employed (*I*^2^ = 43.59%, *P* = .08), resulting in a combined ORR of 14% (95% confidence interval [CI]: 9%–19%). Furthermore, the DCR across these studies ranged from 38% (Arkenau(2018)^[3]^) to 95% (Jin(2023)^[15]^), leading to a pooled DCR of 66% (95% CI: 53%–77%), which also exhibited significant heterogeneity (*I*^2^ = 79.75%, *P* < .001) (Fig. 2).

### 3.4. Survival

All studies incorporated in this analysis reported both PFS and OS metrics, with the exception of the studies conducted by Luis(2021)^[20]^ and Cousin(2022),^[17]^ which did not provide OS data, and Yarchoan(2021),^[19]^ which omitted both PFS and OS measurements.

**Figure 3.**
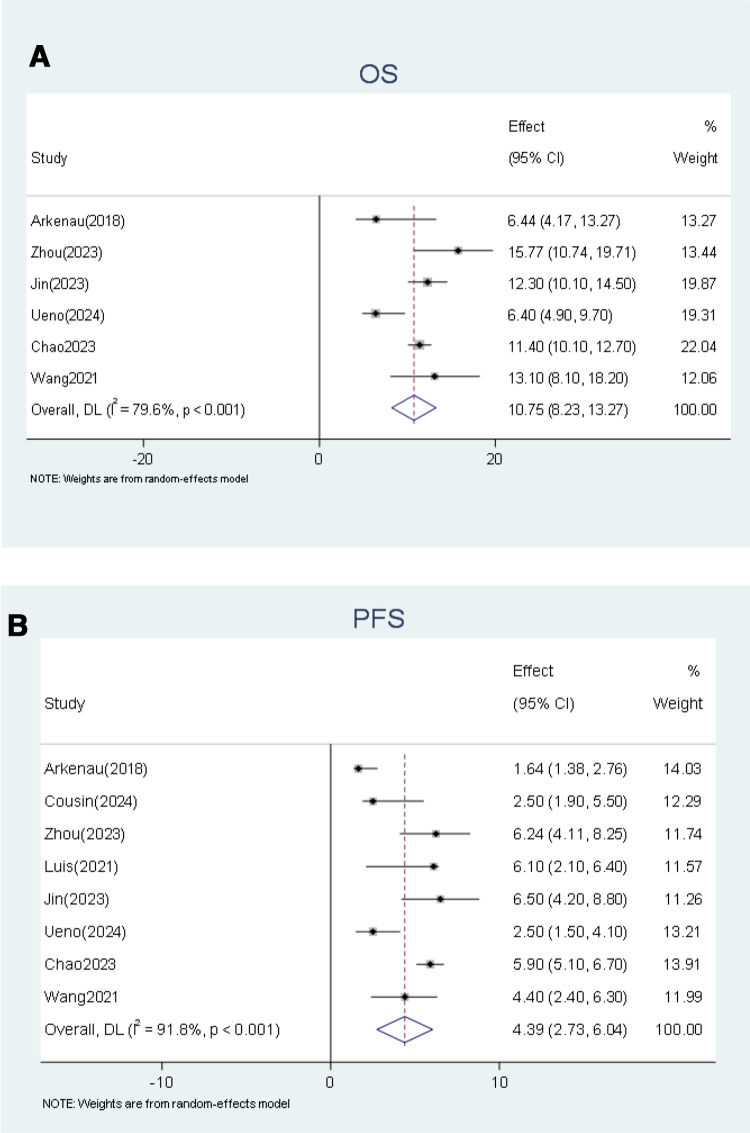
Forest plot about the pooled results of OS (A) and PFS (B) in total by the treatment regimen. OS = overall survival, PFS = progression-free survival.

The OS across these investigations exhibited considerable variability, ranging from 6.40 months to 15.77 months. Employing a random effects model (*I*^2^ = 79.60%, *P* < .001), we computed a pooled median OS of 10.75 months (95% CI: 8.23–13.27 months) as illustrated in Figure 3A. Correspondingly, PFS values ranged from 1.64 months (Arkenau(2018)^[3]^) to 6.5 months (Jin(2023)^[15]^). The analysis of PFS, employing a random effects model (*I*^2^ = 91.80%, *P* < .001), revealed a combined median PFS of 4.39 months (95% CI: 2.73–6.04 months), as depicted in Figure 3B.

### 3.5. Toxicities

AEs, categorized by severity across all grades, including those rated ≥III, were reported in all studies included in this meta-analysis. The majority of patients experienced mild to moderate AEs (Grades 1–2), which were generally well tolerated. Based on incidence rates, we summarized the top ten AEs. Gastrointestinal toxicities were the most prevalent, with elevated aminotransferase levels, decreased appetite, and nausea leading the list. Systemic symptoms were the second most common, comprising fatigue and fever. Cardiovascular, hematological, dermatological, and endocrine toxicities were reported less frequently, each represented by a single event: hypertension, thrombocytopenia, rash, and hypothyroidism, respectively. The analysis identified the 3 most prevalent AEs: aminotransferase increased (incidence 56%, 95% CI: 35%–76%), fatigue (incidence 44%, 95% CI: 31%–58%), and hypertension (incidence 41%, 95% CI: 28%–55%). Notably, the incidence of Grade ≥ III AEs was relatively low, with most events occurring at a rate of ≤ 4%. However, aminotransferase increased, and hypertension was particularly concerning, with incidences of 8% (95% CI: 5%–13%) and 14% (95% CI: 5%–27%), respectively (Table 3).

**Table 3:** Adverse events of the studies included in the meta-analysis.

AE	All grade		≥Grade III	
	ES, (95% CI)	I^2^, %	ES, % (95% CI)	*I*^2^, %
Aminotransferase increased	0.56 (0.35–0.76)	92.20	0.08 (0.05–0.13)	23.57
Fatigue	0.44 (0.31–0.58)	84.04	0.03 (0.01–0.07)	37.64
Hypertension	0.41 (0.28–0.55)	84.05	0.14 (0.05–0.27)	86.33
Diarrhea	0.31 (0.24–0.39)	51.23	0.04 (0.01–0.07)	24.60
Thrombocytopenia	0.31 (0.23–0.39)	51.31	0.04 (0.01–0.08)	28.43
Rash	0.30 (0.22–0.39)	57.22	0.04 (0.02–0.06)	0.00
Hypothyroidism	0.30 (0.20–0.41)	71.61	0.00 (0–0.01)	0.00
Decreased appetite	0.27 (0.14–0.42)	86.96	0.01 (0–0.03)	21.41
Nausea	0.23 (0.18–0.29)	0.00	0 (0–0.01)	0.00
Fever	0.22 (0.15–0.31)	25.13	0 (0–0.02)	0.00

CI = confidence interval.

### 3.6. Sensitivity analysis

A sensitivity analysis was conducted to evaluate the influence of excluding individual studies on the overall outcomes. The results revealed that the pooled estimates within the 95% CI remained largely unchanged with the omission of any single study. This finding underscores the robustness of the conclusions drawn from this meta-analysis.

### 3.7. Publication bias

**Figure 4.**
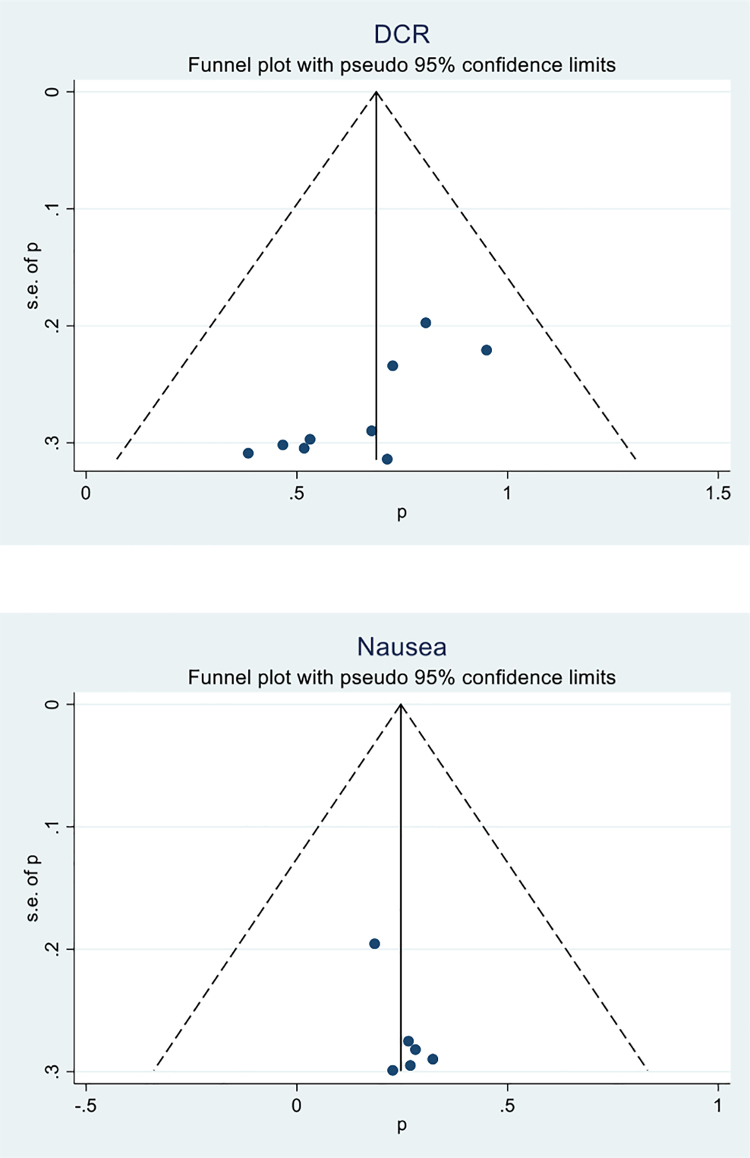
Funnel plot and Egger’s test for assessment of publication bias in the incidence of nausea. DCR = disease control rate.

To ensure the validity of the meta-analysis results, both Egger’s test and funnel plots were utilized to evaluate publication bias. The outcomes of these assessments were largely consistent with the overall findings; however, notable exceptions were identified for the DCR (Egger’s test: *P* = .001) and the incidence of nausea (Egger’s test: *P* = .022) (Fig. 4).

## 4. Discussion

The treatment of advanced BTC poses considerable challenges. According to the ABC-06 trial,^[6]^ regimens based on FOLFOX and irinotecan represent appropriate second-line treatment options; however, there remains substantial room for improvement in overall patient outcomes. The absence of a consensus on alternative treatment strategies following the failure of second-line therapies underscores the necessity for more definitive guidelines in the management of advanced BTC. In response to the pressing need for effective interventions, this study presents the first meta-analysis systematically evaluating targeted combination immunotherapy in patients with BTC who have experienced failure of at least one line of systemic therapy. This analysis establishes a critical foundation for further exploration of this treatment modality, underscoring its potential to enhance clinical practice in the management of aggressive advanced BTC.

The results of this meta-analysis yield critical insights into treatment efficacy and safety. The ORR across the included studies varied from 4% to 33%, yielding a combined ORR of 14%. The pooled DCR was observed at 66%. Furthermore, median OS was recorded at approximately 10.75 months, with a median PFS of 4.39 months. Regarding AEs, the majority of patients experienced only Grade 1 to 2 events, indicating a generally favorable tolerability of the treatment regimen. The most commonly reported AEs included elevated aminotransferase levels (56%), fatigue (44%), and hypertension (41%). Notably, Grade ≥III AEs were infrequent, with rates predominantly below 4%. Among these severe events, hypertension and elevated aminotransferase levels occurred at rates of 14% and 8%, respectively, suggesting that the treatment regimen maintains a relatively positive safety profile. These findings underscore the importance of continued investigation into targeted combination immunotherapy as a promising avenue for improving outcomes in advanced BTC management.

### 4.1. Treatment efficacy

In the ABC-06 trial,^[6]^ the median OS for the ASC + FOLFOX group was 6.2 months, with 6-month and 12-month OS rates of 50.6% and 25.9%, respectively, highlighting the potential benefits of this treatment. However, the ORR was only 5%, indicating limited efficacy. In contrast, the meta-analysis reported an ORR ranging from 4% to 33%, with a total ORR of 14%. Notably, the median OS for combination immunotherapy in the meta-analysis was 10.75 months, significantly surpassing the 6.2 months reported in the ABC-06 study. This suggests that targeted combination immunotherapy may offer greater potential for extending survival. Additionally, the median PFS in the meta-analysis was 4.39 months, slightly higher than ABC-06 study’s 4.0 months, further indicating that targeted combination immunotherapy might be more effective in delaying disease progression.

When evaluating second-line treatment regimens, the Zheng et al.^[24]^ Compared XELIRI to irinotecan monotherapy, revealing that the XELIRI group had a median PFS of 3.7 months and an OS of 10.1 months. In contrast, the irinotecan group reported a median PFS of 2.4 months and an OS of 7.3 months. While the OS for the XELIRI group aligns with the meta-analysis findings, its PFS is notably lower than the 4.39 months observed in the meta-analysis. This discrepancy may stem from differences in patient selection, treatment regimens, and the mechanisms of targeted combination immunotherapy. Furthermore, the DCR for the XELIRI group was 63.3%, closely matching the 66% reported in the meta-analysis, indicating similar efficacy in disease control. However, treatment adherence in the XELIRI group declined significantly throughout the treatment process, presenting challenges in clinical application. This decline was less pronounced in the meta-analysis, likely due to the superior tolerance and flexibility of the treatment regimens associated with targeted combination immunotherapy.

The NIFTY study^[25]^ revealed that the combination of liposomal irinotecan with fluorouracil and calcium leucovorin significantly improved median PFS to 7.1 months. In contrast, our meta-analysis reported a median PFS of only 4.39 months, indicating a need for further investigation into enhanced outcomes. The NIFTY study^[25]^ documented a median OS of 8.6 months. Notably, our meta-analysis found an even higher median OS of 10.75 months, suggesting that targeted combination immunotherapy may yield superior survival outcomes and reinforce the potential advantages of this treatment approach. Furthermore, regarding the ORR, the NIFTY study^[25]^ reported an ORR of 14.8% (95% CI 8.1–23.9) based on independent blinded central review (BICR), with investigator assessment yielding 19.3% (95% CI 11.7–29.1). Our meta-analysis combined for an ORR of 14%, indicating comparable efficacy of the treatment regimens and adding to the body of evidence supporting the effectiveness of therapy combinations. While ORR illustrates the immediate effects of treatment, the DCR provides further insight into long-term efficacy. The NIFTY study^[25]^ reported a DCR of 64.8% (95% CI 53.9–74.7) for the liposomal irinotecan group. Our meta-analysis corroborated this with a pooled DCR of 66%, reinforcing the effectiveness of combination therapies in managing disease progression in BTC. In summary, while the NIFTY study^[25]^ demonstrates significant improvements in PFS and OS with liposomal irinotecan, our meta-analysis suggests that targeted combination immunotherapy may offer comparable or even superior outcomes. These findings position targeted combination immunotherapy for BTC as a promising alternative to traditional chemotherapy regimens, highlighting the need for further research to explore its potential advantages.

### 4.2. Treatment safety

In evaluating cancer treatments, the safety of therapies is as crucial as their efficacy. The ABC-06 trial^[6]^ revealed a 69% incidence of Grade ≥3 AEs in the ASC + FOLFOX group, compared to 52% in the ASC-only group. The primary adverse reactions included neutropenia (12%), fatigue (11%), and infection (10%). Furthermore, 3 treatment-related fatalities were highlighted in the ASC + FOLFOX group, indicating significant risks linked to this treatment regimen. In contrast, our meta-analysis demonstrated that the majority of patients experienced only Grade 1 to 2 AEs, reflecting a generally favorable tolerance for the treatments analyzed. The most frequently reported AEs included increased aminotransferase levels (56%), fatigue (44%), and hypertension (41%), with the incidence of Grade ≥3 events remaining low: most below 4%; the highest rates were hypertension and increased aminotransferase levels at 14% and 8%, considered manageable. Thus, the high incidence of severe AEs in the ABC06 trial suggests significant risks associated with conventional chemotherapy options like ASC + FOLFOX, while the favorable safety profile seen in our meta-analysis supports exploring alternative therapies that may offer better tolerability for cancer patients.

Similarly, in the zheng, et al.^[24]^ patients receiving the chemotherapy regimen XELIRI, composed of irinotecan and capecitabine, exhibited considerable adverse reactions, highlighting significant safety concerns. Specifically, leukopenia affected 26.7% of patients, with 93.3% classified as Grade 3/4. This level of immunosuppression raises serious risks, increasing susceptibility to infections and the potential need for hospitalization. Neutropenia was reported in the same percentage, with 83.3% facing severe forms. Additionally, thrombocytopenia and anemia compounded the clinical complexities, necessitating vigilant management. Gastrointestinal complications, such as severe nausea and diarrhea, further underscore the broader safety challenges associated with XELIRI. Despite no treatment-related deaths being reported, the significant rates of adverse reactions warrant careful monitoring and proactive management to ensure both patient safety and compliance.

Conversely, the NIFTY study^[25]^ evaluated the safety profile of liposomal irinotecan combined with fluorouracil-leucovorin in 88 patients and found specific hematological AEs. Anemia occurred in 6% of patients for Grade 1–2 and 9% for Grade 3, while neutropenia was noted at 18% for Grade 1–2 and 8% for Grade 3. Gastrointestinal events were reported in 26% of participants, including diarrhea, pyrexia, and anorexia. Additionally, the most common Grade 3–4 AEs were neutropenia (24%) and fatigue or asthenia (13%). Notably, serious AEs occurred in 42% of patients receiving the combination treatment, compared to 24% in those receiving fluorouracil and leucovorin alone. Fortunately, no treatment-related deaths occurred, suggesting an overall acceptable safety profile despite the higher rates of serious complications. In conclusion, the findings from the ABC-06 trial,^[6]^ Zheng et al.,^[24]^ and NIFTY studies^[25]^ illustrate distinct safety profiles for cancer treatment regimens. The ABC-06 trial^[6]^ and Zhang et al.^[24]^ highlight significant concerns regarding severe adverse effects that necessitate vigilant management. Conversely, the favorable safety profile of liposomal irinotecan combined with fluorouracil-leucovorin aligns with our meta-analysis findings that immunotherapy and targeted therapies may offer a more stable safety profile, allowing for sustained treatment with fewer interruptions. Overall, understanding the varying safety profiles of these different cancer treatment regimens is critical. Clinicians must carefully weigh the risks and benefits of available treatment options, striving to optimize patient outcomes while minimizing adverse effects, especially in advanced cancer contexts. These insights significantly contribute to clinical decision-making and improve patient care in oncology.

### 4.3. Future directions

With the advancement of gene testing technologies, novel targeted therapies for BTC are continually emerging. For instance, therapeutic agents are being developed for targets such as IDH1 (approximately 10%–20% in iCCA), FGFR2 (9%–15% in iCCA), NTRK (<1%), HER2 (5%–20% in cholangiocarcinoma and 15%–20% in GBC), BRAF (1%–5%), MEK, and microsatellite instability-high (1%–3%).^[26]^ For patients with FGFR2 mutations in iCCA, recommended targeted therapies include Pemigatinib (approved by the FDA/EMA), Derazatinib, Infigratinib (FDA-approved), Futibatinib, and Erdafitinib. Patients with IDH1 mutations may be treated with Ivosidenib and Enasidinib. For those harboring KRAS G12C mutations, which account for 30%–45% of eCCA and GBC cases, KRAS inhibitors may be employed. HER2-targeted therapies include Zanidatamab, combinations of trastuzumab and pertuzumab, trastuzumab, Lapatinib, and Varlitinib.^[27,28]^ In cases of BTC associated with BRAF mutations, a regimen involving Dabrafenib and Trametinib is indicated. Additionally, homologous recombination deficiency, found in 5%–15% of BTC cases, involves genes such as ARIDIA, BAPI, ATM, BRCA2, CHEK2, and PALBB2, making these patients sensitive to platinum-based chemotherapy. Current investigations are assessing the efficacy of combining PARP inhibitors with other treatments.^[27,28]^ Moreover, patients with deficient mismatch repair/microsatellite instability-high (2%–5%) status in BTC are sensitive to ICIs. These advancements in genetic characterization and targeted therapies hold promise for improving treatment outcomes in BTC patients.

Our meta-analysis has important implications for clinical practice. First, targeted combination immunotherapy has significantly improved overall survival and response rates in patients with advanced BTC, suggesting that clinicians should prioritize this emerging treatment paradigm when developing treatment plans for patients who have failed at least one line of systemic therapy. Clinicians need to reassess existing treatment protocols and consider incorporating targeted combination immunotherapy into treatment guidelines to optimize patient outcomes. Additionally, these findings underscore the importance of personalized treatment for patients; when selecting a therapeutic regimen, physicians should take into account the patient’s specific condition, prior treatment responses, and tolerability to formulate the most beneficial treatment strategy. Furthermore, healthcare teams should enhance patient education and support regarding treatment to improve patients’ acceptance and adherence to targeted therapies, thereby maximizing treatment effectiveness.

We acknowledge several limitations that may affect the interpretation and generalizability of our findings. Most included studies were non-randomized and single-arm, increasing the risk of selection bias and confounding; nevertheless, their synthesis represents the most comprehensive current evidence on combination immunotherapy and targeted therapy for advanced BTC, highlighting the urgent need for prospective comparative trials. Treatment regimens were heterogeneous, encompassing various combinations of PD-1/PD-L1 inhibitors with Tyrosine kinase inhibitors, anti-VEGF, or MEK inhibitors, differing in dose and schedule. Follow-up and outcome reporting were incomplete in some studies, with several lacking OS or PFS data. To mitigate these issues, we applied random-effects models, conducted sensitivity analyses, and transparently reported missing outcomes. Nevertheless, our results should be considered preliminary evidence for the potential efficacy and safety of targeted combination immunotherapy. Future studies with larger, more homogeneous cohorts, standardized regimens, comprehensive follow-up, and ideally randomized designs with multivariable adjustments are warranted to validate these findings. Although tumor location may influence treatment response and prognosis, the majority of included studies did not provide outcome data stratified by iCCA, eCCA, or GBC. Therefore, a subgroup analysis could not be performed. Future prospective studies reporting tumor-specific outcomes are needed to enable such analyses.

## 5. Conclusions

This research provides compelling evidence for the efficacy and reliability of targeted therapies and immunotherapies in patients with advanced BTC. These approaches offer valuable treatment options for individuals who experience disease progression after chemotherapy, as well as for those unable to tolerate or who choose to decline chemotherapy.

## Author contributions

**Data curation:** Yiran Liao.

**Formal analysis:** Yiran Liao.

**Methodology:** Yiran Liao, Zhongli Liao.

**Software:** Yiran Liao, Zhongli Liao, Jiong Wang.

**Validation:** Yiran Liao.

**Resources:** Zhongli Liao, Jiong Wang.

**Writing – original draft:** Yiran Liao, Zhongli Liao, Jiong Wang.

**Writing – review & editing:** Yiran Liao, Jiong Wang.
